# Radionuclide Imaging of Fungal Infections and Correlation with the Host Defense Response

**DOI:** 10.3390/jof7060407

**Published:** 2021-05-22

**Authors:** Alfred O. Ankrah, Mike M. Sathekge, Rudi A. J. O. Dierckx, Andor W. J. M. Glaudemans

**Affiliations:** 1 National Centre for Radiotherapy Oncology and Nuclear Medicine, Korle Bu Teaching Hospital, Accra GA-222 7974, Ghana; ankrah.alfred@gmail.com; 2 Department of Nuclear Medicine, University of Pretoria, Steve Biko Academic Hospital, Pretoria 0001, South Africa; mike.sathekge@up.ac.za; 3 Medical Imaging Center, University Medical Center Groningen, University of Groningen, 9700 RB Groningen, The Netherlands; r.a.dierckx@umcg.nl

**Keywords:** fungi, host defence, inflammation, invasive fungal infections, radionuclides, PET, SPECT

## Abstract

The human response to invading fungi includes a series of events that detect, kill, or clear the fungi. If the metabolic host response is unable to eliminate the fungi, an infection ensues. Some of the host response’s metabolic events to fungi can be imaged with molecules labelled with radionuclides. Several important clinical applications have been found with radiolabelled biomolecules of inflammation. ^18^F-fluorodeoxyglucose is the tracer that has been most widely investigated in the host defence of fungi. This tracer has added value in the early detection of infection, in staging and visualising dissemination of infection, and in monitoring antifungal treatment. Radiolabelled antimicrobial peptides showed promising results, but large prospective studies in fungal infection are lacking. Other tracers have also been used in imaging events of the host response, such as the migration of white blood cells at sites of infection, nutritional immunity in iron metabolism, and radiolabelled monoclonal antibodies. Many tracers are still at the preclinical stage. Some tracers require further studies before translation into clinical use. The application of therapeutic radionuclides offers a very promising clinical application of these tracers in managing drug-resistant fungi.

## 1. Introduction

Fungal infections cause high morbidity and mortality, especially in immunocompromised patients [1]. The human body recognises fungi invading the body by pattern recognition receptors. The pattern recognition receptors are genetically determined receptors that recognise molecular sequences associated with fungi. These receptors are different from the receptors of the adaptive immune system formed by T and B lymphocytes in response to an antigenic challenge. Dectin-1 best illustrates the function of pattern recognition receptors in fungal infections. Dectin-1 recognises B-glucan sequences in the inner layer of the cell wall present in all fungi species [2,3,4]. Once the host recognises the fungi, the receptor-pathogen interaction sets intracellular events that result in the formation of chemokines and cytokines that modulate inflammation. These chemokines and cytokines cause white blood cells to migrate to the infection site, leading to the phagocytosis of the fungi, and eventually to the killing and clearing of the fungi [2,5]. Imaging these inflammatory events showed added value in the management of these infections.

Anatomic-based imaging in infection often depends on structural changes that may not occur early in the sequence of the host’s reaction to the fungi. Similarly, after an established fungal infection is treated with antifungal agents, the anatomic changes associated with the infection may persist long after the offending fungi are cleared. The metabolic changes that occur with inflammation due to the host response precede the anatomic changes, and can be used during and after the treatment. These metabolic changes can be imaged with radionuclide-based tracers [6].

Radionuclide imaging includes positron emission tomography (PET) and single-photon emission computed tomography (SPECT), and provides functional data of structures deep within the body. PET and SPECT imaging rely on the radiolabelling of chemical substances that resemble or are actual biomolecules of the body’s chemical processes. The radionuclides emit energy directly as photons or after initially forming positrons. SPECT or PET camera systems image the emitted photons. These cameras allow the three-dimensional acquisition of pathophysiologic processes in the body. PET and SPECT imaging are usually combined with anatomic-based imaging (PET/CT and SPECT/CT). The anatomic-based imaging allows anatomic localisation and attenuation correction (when possible) of PET and SPECT images. PET imaging has better resolution than SPECT, and quantification of radiotracer tissue uptake has been better validated for PET in clinical practice. SPECT is more widely available and relatively cheaper [7].

In this review, we describe radionuclide-based PET and SPECT imaging agents used in inflammation and their correlation to the events of host defence of fungi. Medical imaging plays a vital role in the management of fungal infections [8]. Imaging is part of the diagnostic workup of patients with suspected fungal infection [9]. Imaging is able to stage the infection, to detect occult or disseminated infection, to monitor antifungal therapy, and to provide prognostic information [10,11].

## 2. PET Imaging

PET imaging is performed after the injection of PET tracers into a patient. PET tracers are compounds in which an element is replaced by a radioactive positron emitter. A positron is a positive electron that is not naturally present in nature, but is produced by radioactivity. After a positron is released by radioactive decay, the positron travels a short distance and combines with an electron in the host tissue or environment. The combined positron and electron undergo annihilation, and their mass is converted to energy; the energy is given of as two 511 keV photons travelling in opposite directions. A PET camera system is able to detect the two electrons simultaneously. An image is reconstructed from the photons to provide a three-dimensional representation of the underlying pathologic or physiologic process that the PET tracer that was injected was meant to image.

### 2.1. ^18^F-FDG PET in Fungal Infections

^18^F-Fluorodeoxyglucose (^18^F-FDG) is a glucose analogue and the most common PET tracer in the clinical setting. ^18^F-FDG is widely used as the radiolabelled agent of choice in imaging many infections and inflammatory conditions [12,13,14,15,16]. It has been used in the evaluation of different aspects of fungal infections. After fungi invade the body’s tissues, they are recognised by pattern recognition receptors which, in turn, initiate intracellular signals that result in inflammation to clear the fungi. The inflammatory processes that are activated use energy. These energy-dependent processes result in the uptake of glucose by the immune cells. The increased glucose uptake by the GLUT transporters on the cell membrane of immune cells generates a signal with ^18^F-FDG. This forms the basis of imaging inflammation with PET [17]. ^18^F-FDG PET is usually integrated with computed tomography (CT). Recently, some systems also integrate PET with magnetic resonance imaging in clinical practice. The positron ^18^F has a half-life of 110 min, allowing enough time for the tracer to be taken up in the inflamed tissue and to be imaged by PET, while not lasting too long in the host to give an unnecessarily high radiation burden.

Several case reports and case series with relatively small numbers documented the ability of ^18^F-FDG PET/CT to detect lesions of fungal infections early before anatomic changes, the usefulness in detecting occult lesions and detecting dissemination, and the ability to monitor antifungal therapy [18,19,20,21,22]. The role of ^18^F-FDG PET/CT was further confirmed in several larger studies [23,24,25,26,27].

**Figure 1:**
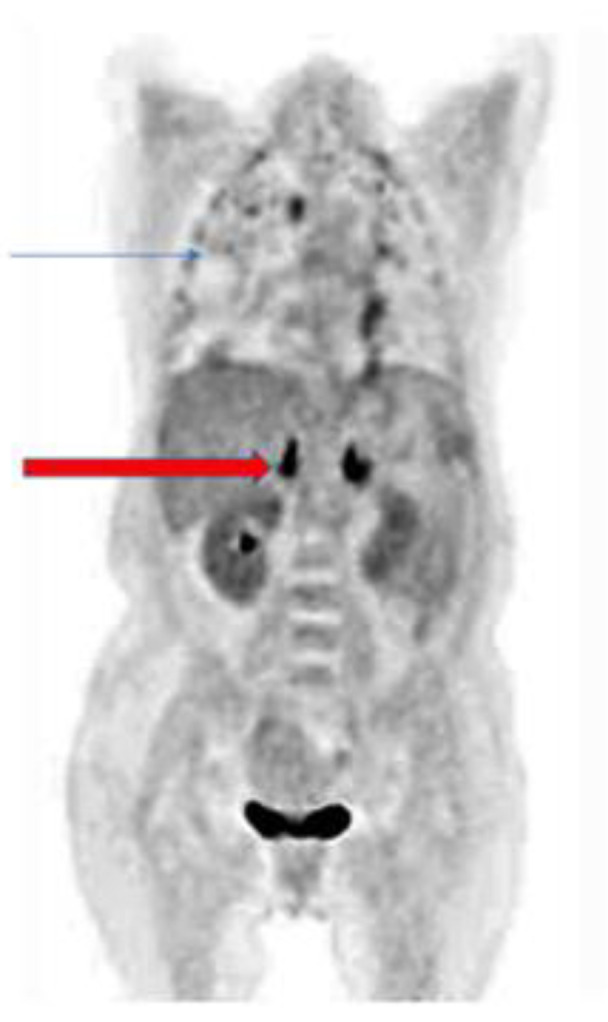
Patient with pulmonary cryptococcosis (blue arrow) with bilateral intense hypermetabolic uptake on coronal slice of ^18^F-FDG PET due to adrenal involvement (red arrow).

The pattern of uptake may direct the imaging specialist to the presence of a fungal infection. Bilateral adrenal gland uptake, for example, may occur in *Cryptococcus* or *Histoplasmosis sp.* infections. Figure 1 shows a patient with *Cryptococcus neoformans* with bilateral adrenal gland uptake due to the fungi [20,28,29,30,31]. Knowledge of the epidemiology of fungal infection and the patterns that may occur is important in the interpretation of ^18^F-FDG PET/CT in fungal infections.

There are numerous species of fungi that may cause an invasive fungal infection. In the literature, most of the case reports and studies focus on radionuclide imaging of *Candida sp*. and *Aspergillus sp.* because these are often the most common species in blood-stream infection and infections among the immunocompromised, such as patients with hematologic malignancies. ^18^F-FDG PET/CT has been shown to be useful in the diagnosis and monitoring of treatment in other fungi such as Histoplasmosis sp. *Cryptococus neoformis* and *Pneumocystis jeroveci pneumonia* [19,28,29,30].

### 2.2. Other PET Tracers Used for Inflammation and Their Role in Fungal Infection

#### 2.2.1. PET Labelled Siderophores

One method of defence by the human body from pathogens is to ensure that the nutrients needed for the survival of the microorganism are not available to the pathogen. These nutrients, such as iron or copper, are highly regulated by being bound to various molecules, so they are not readily available to pathogens. This is called nutritional immunity. To overcome this, fungi and other pathogens adopt various mechanisms [32]. Fungi employ siderophores to overcome the relative deficiency of iron caused by the host nutritional immunity. Iron is an important cofactor for different physiologic processes, such as respiration, metabolism of amino acids and biosynthesis of nucleic acids and steroid compounds [33,34]. Siderophores tend to be species-specific. More than 500 different structures of siderophores have been identified among fungi, bacteria, and other microorganisms [35]. Some siderophores are endogenous in the pathogens, while others are secreted outside the pathogen. *Aspergillus fumigatus* secretes two siderophores—fusarinine C and triacetylfusarinine C—in addition to two endogenous siderophores it possesses [36,37]. In fungi, siderophore–iron transporters in the fungal membrane transport secreted siderophores which have chelated iron from the host into the fungal cell. Siderophore–iron transporters occur in most fungal species, and this transporter is only found in fungi [37,38]. These factors make secreted siderophores a good substrate to target fungi. Siderophores have been labelled with PET radioisotopes, which are analogues of iron: Gallium 68 (^68^Ga) with a half-life of 68 min, and Zirconium 89 (^89^Zr) with a half-life of 78 h [39,40]. PET imaging with ^68^Ga has gained prominence recently with GMP-grade Germanium 68/Gallium 68 generator availability [41]. The short half-life of 68 min gives ^68^Ga favourable radiation burden characteristics compared to the longer-lived ^89^Zr. The PET tracers ^68^Ga or ^89^Zr as iron analogues are chelated with siderophores as a complex and injected into the patient. The siderophore–iron analogue complex is transported into the fungi by the siderophore–iron transporters and allow the fungi to be imaged. These labelled siderophores demonstrated a high uptake in *Aspergillus fumigatus*-infected lungs with high target to nontarget ratio in rodent models. The potential translation of radiolabelled siderophores to the clinic is limited by the differences in uptake among other fungal and bacterial species. There are research studies ongoing to modify siderophores, to make them more acceptable as a PET agent for infection [40,42].

#### 2.2.2. PET Labelled Antibodies

Antibodies produced by B lymphocytes are part of the host immune responses against fungi. The specific contribution of antibodies to the host defence against fungi has been a contentious issue. Earlier investigators suggested antibodies were not necessary for host defence against fungi in humans [43,44]. The use of monoclonal antibodies has helped advance our understanding of antibodies’ vital role in different fungal species [45,46,47,48]. The use of an agent that targets a specific molecule present on the pathogen in a similar way to a “magic bullet” offers a desirable method for targeting microbes or molecules expressed in pathophysiologic processes in the body. The combination of PET labels to antibodies targeting specific molecules in patients is referred to as immunoPET. ImmunoPET has been used extensively in oncological applications. There is an increased interest in the use of immunoPET in infectious diseases [49]. A monoclonal antibody JF5 has been developed and labelled with Copper 64 (^64^Cu) with a half-life of 12.7 h [50,51]. The longer half-life of ^64^Cu (compared with ^18^F) is important, as antibodies and high molecular weight molecules require a longer time to clear from the plasma. A more efficient way of producing ^64^Cu from a solid rather than liquid target has made the use of ^64^Cu more favourable [52]. The JF5 radiolabel can differentiate actively growing hyphae of *Aspergillus sp*. from inactive spores. JF5 binds to galactofuranose glycoprotein, which is expressed on the hyphae in medically important species of *Aspergillus*. Both the murine antibody and humanised antibody are currently available [50,53,54]. There is no non-specific binding of JF5 to mammalian tissues as the epitope β1,5-galactofuranose is not present in mammalian tissues. The ^64^Cu-JF5 showed good tracer accumulation in the lungs in invasive pulmonary aspergillosis in an animal model [50].

#### 2.2.3. PET Labelled Antifungal Agents

Antifungal agents help combat fungal infections when the host defence is unable to clear invading fungi resulting in an infection. The antifungal agent fluconazole has been labelled with ^18^F. Fluconazole is a synthetic azole that inhibits ergosterol biosynthesis. Ergosterol is a lipid that is required in the synthesis of the fungal cell membrane. ^18^F-fluconazole was studied in preclinical animal models of candidiasis. Unfortunately, the tracer showed high renal uptake, and the uptake in tissues with fungal infection was not impressive [55,56,57].

Recently, the antifungal drug amphotericin B has been labelled with ^68^Ga in an in vitro model. This radiotracer showed good accumulation in inserts with clinically important moulds such as *Aspergillus fumigatus*, and *Rhizopus arrhizus*. These results must be tested in animal models of fungal infections [58].

#### 2.2.4. Gallium 68 (^68^Ga) Citrate

^68^Ga citrate is a PET tracer that has been evaluated in infection and inflammation [59,60]. Its corresponding SPECT tracer Gallium (^67^Ga) citrate was used for inflammation and infection in the past, but that role has been largely supplanted by ^18^F-FDG. Gallium is an iron analogue, and the body iron binding proteins in the body handle it in a similar way to iron [61]. The role of iron and host nutritional immunity has been earlier described. Although the SPECT tracer (^67^Ga citrate) was used in fungal infections, especially in HIV patients in the past, ^68^Ga citrate is yet to be evaluated in fungal infections. One area it may potentially play a role in is intracranial lesions, where the accumulation of ^18^F-FDG in the brain may limit detection of intracranial pathology with ^18^F-FDG. In *Mycobacterium tuberculosis*, ^68^Ga citrate showed better lesion detection of intracranial lesions, due to the absence of tracer accumulation in the brain [62]. Moreover, studies have suggested ^68^Ga citrate is less likely to accumulate in post-infective lesions after treatment of the different infections [62,63,64]. Large multi-centred prospective studies are needed to investigate the use of ^68^Ga citrate in fungi for these indications.

#### 2.2.5. PET Labelled White Blood Cells

As part of the host response to fungi, cytokines attract white blood cells to the site of infection in the tissues. Radiolabelled white blood cell imaging using SPECT tracers is the only nuclear medicine imaging method that is able to differentiate between infection and inflammation [65,66]. The labelling of white blood cells with radionuclides is by drawing blood from the patient, separating white blood cells, radiolabelling and re-injecting them [67]. White blood cells have also been labelled with ^18^F-FDG to take advantage of the better resolution and image quantification of PET imaging [68]. However, the labelling efficiency of ^18^F-FDG was not as good as the SPECT tracer imaging methods for labelling white blood cells. Furthermore, the half-life of ^18^F (110 min) is not favourable for delayed imaging, and normally it takes around 20 h for the white blood cells to accumulate at the site of infection [69]. Therefore, white blood cells have been labelled with ^64^Cu and other longer-lived PET radioisotopes to allow for delayed imaging with PET [70,71]. White blood cell labelling has the disadvantage of the need to handle blood products. This carries the risk of transmission of blood-borne infectious disease to the staff involved in the labelling of the blood products and to the patients whose white cells are being labelled [72]. Again, some patients with fungal infections may be critically ill or also suffer from neutropenia; labelling of white blood cells under these circumstances may not be practical. Furthermore, there have been concerns about the radiation damage to the lymphocytes by the labelled radionuclide [72,73]. These factors have not allowed PET-labelled white blood cells to supplant their counterpart in infection imaging. The difficulty of labelling white blood cells in patients with fungal infection makes ^18^F-FDG PET the preferred method at this moment.

#### 2.2.6. PET Labelled Antimicrobial Peptides

Antimicrobial peptides are small molecular weight cationic peptides released by immune cells to kill fungi and other pathogens as part of the innate immune response [4]. Fragments of antimicrobial peptides such as ubiquicidin (29–41) have been labelled with ^68^Ga [74,75]. This peptide is expected to accumulate in *Aspergillus fumigatus* and *Candida albicans* and bacteria similarly to its SPECT counterpart, as the mechanism of uptake is the binding of the cationic peptide to the negative charge on the membrane of the microbe for both PET and SPECT tracers. The antimicrobial peptides lack a tertiary structure; hence, the different labelling methods, in the same way as the different methods used for labelling PET or SPECT tracers, may affect their ability to target and image microorganisms. Studies of the PET-labelled antimicrobial peptides are needed to see if it will be as efficient in fungi as its SPECT counterpart.

## 3. SPECT Tracers in Inflammation

SPECT imaging is based on the detecting photons that usually arise from a radioisotope nucleus by radioactive decay [76]. The most used radioisotope is Technetium 99 m (^99m^Tc), which emits a photon with 140 Kev energy and has a half-life of 6 h. Other frequently used radioisotopes are Indium 111 (^111^In) and Gallium 67 (^67^Ga), with relatively longer half-lives and a significantly higher radiation burden on patients when used for imaging.

### 3.1. SPECT Labelled White Blood Cells

White blood cells labelled with technetium 99m (^99m^Tc) (as ^99m^Tc-hexamyethylpropyleneamine) or indium 111 (^111^In) (as ^111^In oxine) is the preferred nuclear imaging method of choice to differentiate infection from inflammation [66]. There are relatively few reports of the use of SPECT-labelled white cell studies in fungal infections [77]. Fungal infections have been noted to cause false-negative white cell studies due to the relative low accumulation of neutrophils compared to other infections [78]. Other agents such as ^18^F-FDG are preferred in imaging fungal infections.

### 3.2. Anti-Granulocyte Imaging

Monoclonal anti-granulocyte antibodies against granulocytes have been labelled with SPECT-based tracers to overcome the need to handle blood products [79,80,81,82,83]. There are several monoclonal anti-granulocyte antibodies targeting different antigens. The antigens include NCA, CD15, or CD66, which have been tested against infectious agents [76,79,80]. There are only two commercial agents available [66]. No large studies involving fungal infections are available, but one study found the evaluation of pulmonary fungal disease, especially pneumocystis, particularly disappointing [84].

### 3.3. ^67^Gallium Citrate

^67^Ga citrate is a SPECT tracer that has been used extensively in the past in infection and inflammation. The tracer is taken up at infection sites by specific and nonspecific mechanisms. The nonspecific mechanisms include the increased tracer uptake due to increased vascularity because of inflammation. The specific mechanisms are related to increased iron-binding proteins such as lactoferrin, transferrin and siderophores from the pathogen. ^67^Ga has a half-life of 78.3 h. The tracer gives a high radiation burden compared to ^99m^Tc-based imaging, and after injection the patient must wait for about 24 h before imaging. The tracer was also nonspecific accumulating in noninfective lesions [85]. These factors led to the search for a better imaging agent, and ^18^F-FDG has taken over most of the indications ^67^Ga previously had. ^67^Ga citrate was important in the evaluation of fungal infection, especially in HIV patients [86,87].

### 3.4. SPECT Labelled Fluconazole

The antifungal drug fluconazole has been labelled with ^99m^Tc. This tracer accumulated in sites infected with *Candida albicans* in a mouse model [88,89]. It did not accumulate in bacterial infection nor in sterile inflammation. The intensity of tracer accumulation correlated to the live fungi in infected tissue, suggesting it will be useful in monitoring antifungal therapy. The tracer performed poorly in imaging *Aspergillus fumigatus.* Furthermore, resistance to the antimicrobial may result in false positive results. The intensity of uptake in affected tissue was not high [61]. The agent has not been translated to humans.

### 3.5. SPECT Labelled Amphotericin B

Amphotericin B is another antifungal agent labelled with ^99m^Tc with good results in in vitro studies. Amphotericin B binds irreversible to ergosterol, disrupting the integrity of the cell wall leading to death. The radiolabelled tracer accumulated in Transwell inserts infected with *Aspergillus fumigatus* and other relevant moulds, but not in a bacteria-infected insert. Preclinical studies of this radiolabel in an animal model would move this tracer nearer the potential clinical translation [62].

### 3.6. SPECT Labelled Antimicrobial Peptides

Many antimicrobial peptides have been investigated as potential agents to be labelled with ^99m^Tc, including ubiquicidin fragments and human lactoferrin peptide [88,90,91,92]. The ubiquicidin 29–41, a fragment of the antimicrobial peptide ubiquicidin, has been labelled with ^99m^Tc and been tested in animal model and humans. The labelled fragment accumulated in *Candida albicans, Aspergillus fumigatus* and bacteria. The tracer did not accumulate in sterile inflammation or sites with killed bacteria.

### 3.7. SPECT Labelled Chitin Related Proteins

Chitin is an essential component of the fungal cell wall. In inflammation, different molecular sizes and concentrations of chitin affect fungal immunity differently. Depending on the size, the chitin polymer may induce an inflammatory response or an anti-inflammatory response, or may not evoke any inflammatory response [93]. SPECT-based labels of a chitin-binding protein and the enzyme chitinase have been evaluated for imaging fungal infections [94,95]. In a mouse model, ^123^I-chitinase showed accumulation in mice tissue infected with fungus but not in bacteria or sterile inflammation. A chitin binding protein (CBP21) also showed more accumulation in tissues infected fungi compared to bacteria. The uptake by these agents was modest, with reported target to background ratio higher than 1. No translation to humans of these agents has been done.

### 3.8. Other Radionuclides for Inflammation

Other components of the immune system, such as cytokines IL-1, IL-2, IL-8, and platelet factor 4 have been investigated as agents for imaging infections. Some of these have been unsuccessful due to biological activity of the radiolabel such as induction of fever by IL-1. In some other cases, the labels have found applications in other causes of inflammation but not in infection [96].

Some tissues or cells which are considered not to be a part of the immune system are involved in the host response against fungi. These include epithelial tissues, platelets, and endothelial cells. The epithelial cells express protein recognition receptors that recognise fungi and initiate host response against fungi. The integrity of epithelial tissues is essential in host defence against fungi. The lungs are the most common site of fungal infections; radiolabelled agents can assess the respiratory epithelium’s integrity. In the past, the respiratory epithelial transport has been assessed using inhaled epithelial lung clearance. This procedure is not performed routinely in clinical practice [86].

## 4. Application of Radionuclide Therapy in Host Defence

The labelling methods that target the fungi can be exploited for radionuclide therapy of these agents. Radionuclide therapy delivers damaging radiation to the infected or diseased tissue with minimum damage to the surrounding normal tissue. The radionuclides used for therapy are either alpha or beta particles. An alpha particle is an atomic nucleus containing two protons and two neutrons (a helium nucleus) that is emitted during radioactive decay. The charge of the two protons (2+) and the large size of the two proteins allows it to deliver most of its energy, which often leads to damage to nucleic acid material and other biomolecules killing or inhibiting the growth of cells including fungi in the vicinity of the targeted tissue. Beta particles consist of electrons released with high kinetic energy and also deposit the surrounding tissue. The relative lower mass and lesser charge of the beta particle (1-) compared to the alpha particle lets them deposit less energy compared to alpha particles, but the beta particles are able to deposit their energy over a longer range of tissue. In targeting fungi, the radionuclide attached to the biomolecule for imaging is replaced with a therapeutic radionuclide to target, kill or inhibit the growth of fungi [97]. The targeted radionuclide therapy works on the principle that the side effects are minimal, as radioactive agent is directly delivered to diseased tissues, sparing uninfected tissues. In cases of a widespread infection, however, radionuclide therapy may cause bone marrow suppression, as occurs in other radionuclide therapy, which can be predicted by the diagnostic scan [98].

There are relatively few antifungal agents available, and drug resistance against these antifungal agents is a constant challenge. The use of radioimmunotherapy in fungal infection has been tested in some animal models with encouraging results [99,100,101,102,103]. The radionuclides such as Bismuth 213 (an alpha particle) or Rhenium 188 (a beta particle) are examples of some of the labels used for therapy. Radioimmunotherapy is not subject to resistance in the same way as traditional drugs, and is independent of the patient’s immune status. Monoclonal antibodies against antigens in all fungi such as β-glycan would eliminate the need to develop or know the species before administering fungal radioimmunotherapy [97].

## 5. Clinical Translation of Fungal Agents

Radionuclide imaging of fungal infection has been used for imaging different aspects of the host response to fungi. PET imaging with ^18^F-FDG is the only radionuclide so far that has a major role in imaging the host response to fungi with wide clinical acceptance. Some promising agents are at various stages of production. Table 1 outlines the different agents and whether they have been used in humans. The clinical usage and widespread applicability depends not only on the success of the agents in imaging of fungal infections but also on the demand, ease of preparation and widespread applicability. It is likely that an imaging agent that can really distinguish between infection and inflammation and with a broad spectrum activity against different pathogens may gain wider acceptance than the specialised tracers which are limited to special centres [104].

## 6. Conclusions

The host defence mounted by the body against fungi affords the opportunity for radionuclide imaging with important clinical applications. However, so far, only ^18^F-FDG has widespread acceptance for clinical use in patients with fungal infections, for detection, staging, and therapy evaluation. Other radionuclides have been evaluated only preclinically, or are only used in small patient series. Large multi-centred prospective clinical trial must be performed to evaluate the role of these specific radionuclides in imaging fungal infections.

## Figures and Tables

**Table 1:** Tracers used in imaging fungal infection and whether they have been translated to humans.

Tracer [Ref]	PET or SPECT Imaging	Label	Used in Humans	Aspect of Host Response
FDG [23,24,25,26,27]	PET	^18^F	Yes	Glucose utilisation for energy-dependent processes of inflammation
White blood cells [65,66,67,68,69,70,71]	Both	^18^F, ^64^Cu, ^111^In, ^99m^Tc	Yes	Migration of white blood cells to the site of infection
Monoclonal anti-granulocytes [76,77,78]	SPECT	^99m^Tc	Yes	Migration to white blood cells
Antimicrobial peptides [74,75,86,90,91,92]	Both	^99m^Tc, ^68^Ga	Yes	The cationic peptide reacts with the negative charge on the fungi membrane. Release by cells to kill fungi
Siderophores [39,40]	SPECT	^68^Ga, ^89^Zr	No	Traps iron for fungi to overcome host nutritional immunity
Monoclonal antibodies against fungi	PET	^64^Cu, ^89^Zr	No	Against an antigen JF5 in *Aspergillus fumigatus*
Chitin proteins [92,93]	SPECT	^123^I, ^99m^Tc	No	Chitin has different effect on host response depending on the size
Fluconazole [57,86,87]	Both	^99m^Tc, ^68^Ga	No	Anti-fungal used to combat fungi when host defence fails. Affects ergosterol biosynthesis
Amphotericin [58]	Both	^99m^Tc, ^68^Ga	No	Anti-fungal-irreversible bind to ergosterol
